# Cervical cancer screening with clinic-based Pap test versus home HPV test among Somali immigrant women in Minnesota: a pilot randomized controlled trial

**DOI:** 10.1002/cam4.429

**Published:** 2015-02-04

**Authors:** Barrett Sewali, Kolawole S Okuyemi, Asli Askhir, Jerome Belinson, Rachel I Vogel, Anne Joseph, Rahel G Ghebre

**Affiliations:** 1 Program in Health Disparities Research, University of Minnesota Medical SchoolMinneapolis, Minnesota; 2 Department of Family Medicine and Community Health, University of Minnesota Medical SchoolMinneapolis, Minnesota; 3 Somali Health SolutionsMinneapolis, Minnesota; 4 Cleveland Clinic Women's Health Institute, Preventive Oncology International Inc., Cleveland HeightsCleveland, Ohio; 5 Biostatistics and Bioinformatics Core, Masonic Cancer Center, University of MinnesotaMinneapolis, Minnesota; 6 Department of Medicine, University of Minnesota Medical SchoolMinneapolis, Minnesota; 7 Division of Gynecologic Oncology, Department of Obstetrics and Gynecology, University of MinnesotaMinneapolis, Minnesota

**Keywords:** Cancer screening HPV self-collection test, Pap test, Somali women

## Abstract

Cervical cancer is more common in the Somali immigrant population than the general population in the United States (US). There are low rates of cervical cancer screening among Somali women. This study compares cervical cancer screening test completion rates for a home human papilloma virus (HPV) test and standard clinic Pap test. Sixty-three Somali immigrant women aged 30–70 years who had not undergone cervical cancer screening within the past 3 years were randomly assigned to a home HPV test group (intervention) or a clinic Pap test group (control). Test completion rates were measured at 3 months. Univariate and multivariate logistic regression models were used to explore factors associated with test completion (intention-to-treat analysis). Participants in the HPV test group were 14 times more likely to complete the test compared to those in the Pap test group (*P* = 0.0002). Women who reported having friends/family members to talk about cancer screening were approximately three times more likely to complete any screening test than those who did not (*P* = 0.127) and participants who reported residing in the US longer were more likely to complete a screening test (*P* = 0.011). Future research should explore the potential of using the home-based HPV test kits as an initial approach to cervical cancer screening. Impact: The use of a self-sampling HPV kit has the potential to increase cervical cancer screening in under-served communities in the US.

## Introduction

Significant gains have been made in cervical cancer screening over the past three decades in the United States (US); however, cervical cancer continues to cause morbidity and mortality among immigrant women 1,2. The current literature shows that some racial and ethnic groups in the US have disproportionately higher cervical cancer incidence and mortality rates that is associated with low use of screening services 3,4. One group is US immigrants whose cancer screening rates are far below the national goals 5,6. Studies have shown that more recent immigrants (<10 years) are less likely to screen for cervical cancer compared to women born in the US 7–9.

Cervical cancer is the second most frequent cancer among Somali women between 15 and 44 years of age 10. Harcourt and colleagues showed that among African immigrant women in Minnesota, Somali women were least likely to undergo screening for cervical cancer 11. Other studies by Morrison et al. showed that cervical cancer screening prevalence and adherence among Somali women was below the state and national goals 12,13. To date, several well-established barriers to cervical cancer have been documented in the Somali community and many communities worldwide 14–18.

Human papilloma virus (HPV)–DNA testing in cervical cancer prevention programs has been a topic at the forefront of cervical cancer policy discussions in recent years in developing countries 19,20. HPV–DNA testing provides a novel and alternative pathway to increasing cervical cancer screening among women who may not readily access a clinic Pap test 21. It is important to note that that a self-collected sample is equivalent in sensitivity to a direct collected specimen 22–25. With this new development, there is a growing need to examine preference, acceptability and reach among women who are less likely to access the clinic-based Pap test. Currently in the US, HPV–DNA testing is used in conjunction (cotesting) with the traditional clinic-based Pap test; however, there is growing evidence that use of HPV–DNA testing alone as a primary cervical cancer screening test may be an alternative in resource-limited regions 19,21,26,27, where significant barriers to access clinic-based Pap tests exists and substantial loss to follow up which cripples the effectiveness of cervical cancer screening programs 28–31. Furthermore, the HPV home test kit is growing in use in developing countries and has shown tremendous progress in identifying women at risk of cervical cancer and thus reducing mortality 27,32. There is growing evidence as to the test's sensitivity and efficiency rendering it as an alternative to the traditional Pap test. These findings suggest that primary HPV testing merits consideration as another alternative for cervical cancer screening 33,34.

Given that more than half of cervical cancer deaths in the US are among immigrants, and the incidence and mortality from cervical cancer are increasing among foreign-born women living in the US, 6 understanding the factors that influence women's cervical cancer screening practices and examining which screening options are acceptable in different populations may be an effective way to reduce the existing cervical cancer screening disparities. The objective of this pilot study was to examine the difference in successful test completion rates between home-based HPV tests and clinic-based Pap tests among a sample of Somali immigrant women residing in the Minneapolis/St. Paul area, to see if this innovative testing method might improve cervical cancer screening rates in this particular underserved population.

## Methods

This work was a result of a partnership between a community-based Somali community organization; Somali Health Solutions and the University of Minnesota. Prior to the initiation of the study, community and university research partners met to develop culturally acceptable study informational materials. Using a community-based participatory approach similar to one used by Belinson and colleagues 35; Somali community health workers (CHWs) were trained by University researchers on the study protocol. The protocol training specifically included information on cervical cancer screening guidelines, randomization and a step-by-step instructional guide on use of the home-based HPV kit (“Just For Me,” Preventive Oncology International, Cleveland Heights, Ohio). Study procedures were approved and monitored by the University of Minnesota Institutional Review Board.

### Study design

We planned a two-group randomized controlled pilot study of 64 Somali adult women designed to assess the completion rates of a home-based HPV kit versus standard of care clinic-based Pap test within a 3-month follow-up period. We hypothesized that women offered the HPV—DNA test kit would have a higher test completion rate compared to those in the clinic-based Pap test group.

### Recruitment and randomization

Recruitment started in November 2013 and was completed in February 2014. Screening for study eligibility was conducted by Somali CHWs who were able to speak both Somali and English. The Somali Health Solutions team recruited participants by word of mouth and flyers. Prior to assessing eligibility and obtaining informed consent, we held an hour long informational meetings in an informal setting such as at one of the Somali women's homes or at a community center identified by the Somali Health Solutions staff. Ten to 20 women attended each informational session. These sessions focused on providing information about the home HPV test kit and the clinic Pap test. All participants were provided with written printed materials during the informational sessions. Following the training, all participants at the training completed a survey to determine study eligibility. Participants who were eligible and wanted to participate in the study provided informed consent and medical release forms for their primary care clinics to obtain potential Pap test data. Participants were then randomly assigned in a 1:1 ratio to the clinic based Pap test versus the home-based HPV test using permuted block randomization with varying block sizes. The study statistician provided the CHWs with randomization assignments in sealed envelopes. Each envelope was opened only after the participant was eligible and had provided a signed consent. Participants were compensated with a $25 gift card for their time.

### Participants

This study recruited women of Somali origin, aged 25–70 years, who lived in the US for 10 years or less and who reported not having had a Pap test in the last 3 years. Women with a self- reported past history of a total hysterectomy, cervical cancer, and/or active history of cervical dysplasia were excluded.

### Study procedures

All participants received education materials on HPV and Pap screening tests. The information provided was similar to that currently used by the Minnesota Sage Program and adapted in the study for Somali women. Following certain eligibility criteria, the Sage program provides free or subsidized office visits for breast and cervical exams, as well as mammogram and Pap tests 36.

### Home-based HPV kit group

Participants randomized to the home-based HPV kit group were given a kit to perform their own vaginal HPV sample collection with detailed written instructions. Text and illustrations were translated and tailored for the Somali community. Participants were asked to complete specimen collection and return the sample to the CHWs within 3 months of study enrollment. Participants were provided with a study phone number in case of any questions pertaining to the test. Three months after the study, women in this group were contacted and provided with information by the CHWs to follow up with a Pap test, regardless of whether they completed home HPV testing. Participants were provided with a letter explaining their participation in the study and a request to the clinic to follow up with a clinic-based Pap test as it is the current standard of care. The self-collected samples were placed on specimen cards contained in the kits that were in turn sent to BGI Clinical Laboratories (BGI Shenzhen, Shenzhen, China), certified by National Health and Family Planning Commission of the People's Republic of China for analysis. They were analyzed using SEQHPV, a new validated high-risk HPV genotyping assay based on next-generation genomic sequencing 25.

### Standard clinic Pap group

Participants randomized to clinic pap group (standard of care) were asked to follow up with their established clinic for a Pap test within 3 months of enrollment. Women without insurance or those lacking an established care clinic were given a list of Minnesota-based clinics which have a cancer screening Sage program close to their place of residence.

### Measures

The primary outcome was the successful completion of the assigned screening test within a period of 3 months after the enrollment date. In the clinic Pap test group, completion was defined by documentation of a Pap test result by their primary care clinic during the study time frame. For the home-based HPV collection group, completion was defined as return of the HPV DNA self-sampling kit by the patient to the CHWs within 3 months of study enrollment.

### Surveys

Baseline and other outcome study data were collected using paper surveys administered by the CHWs.

#### Baseline survey

Socio-demographic data collected included age, sex, marital status (single/married/widowed/divorced), need for an interpreter, use of emergency room services, health care provider preference, history of pregnancies, total annual household income (<$25,000, $25,000–99,999, or $100,000 and more), educational level (no formal education to greater than high school), and preference of receiving and sending medical test results.

#### HPV home test acceptability survey

An acceptability survey was conducted among participants who returned the home vaginal-based kit. This survey was conducted on receipt of a completed test from the participants. The test assessed participant's ease to do the test, difficulties encountered to do the test and any concerns they had while collecting the sample.

### Data analysis

Demographic characteristics were summarized as means and standard deviations (SD) for continuous variables and percent in each category for discrete variables. Screening and baseline characteristics were compared by randomization group to check balance due to the small sample size. The primary analysis used an intention-to-treat approach and compared the proportions of successful completion of cervical cancer screening within 3 months of study entry between the clinic-based Pap group and home-based HPV group using a Pearson chi-square test. In addition, univariate and multivariate logistic regression models were conducted to explore factors associated with screening completion. Factors were included in the multivariate model as potential confounders if they were associated with randomization group and/or screening completion (*P* < 0.10). Analyses were conducted using SAS version 9.3 (Cary, NC) and *P* < 0.05 were considered statistically significant.

## Results

**Figure 1:**
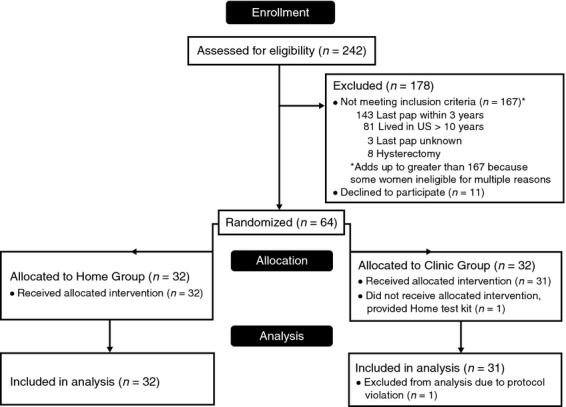
Participant flow chart. *One participant in the clinic group was assigned to clinic but given kit and returned it. This person was removed from the analysis. **There are two additional kits from the Home group that were returned after the 3-month time frame; their outcomes are not included in this analysis.

Of the 242 women screened for the study, 75 (31%) were eligible and 11 declined to participate (Fig.1). Most women who attended the screening sessions were ineligible based on several reasons; a recent Pap smear (within 3 years), unknown Pap history, and history of hysterectomy. A total of 64 women were enrolled and randomized; 32 to the clinic-based Pap group and 32 to the home-based HPV group. One participant in the clinic-based Pap group was removed from analysis due to a protocol violation.

The average age of the 63 participants included in the analysis was 55.1 years (SD = 13.4), the majority were married, had no formal education, primarily spoke Somali, and had an annual household income of less than $25,000 (Table1). There were no significant differences in the demographic characteristics of participants between both groups.

**Table 1:** Baseline characteristics (*n* = 63)

Characteristic	Clinic-based Pap group		Home-based HPV kit group		*P*-value
	*N*	%	*N*	%	
Age mean (SD)	31	54.2 (11.4)	32	56.0 (15.3)	0.585
Years in US, mean (SD)	31	11.2 (5.7)	32	11.8 (5.5)	0.683
Years in MN mean (SD)	30	9.9 (5.6)	31	9.7 (5.6)	0.907
Marital status					
Single/never married	2	6.3	4	12.9	0.119
Married	13	40.6	9	29.0	
Separated	4	12.5	3	9.7	
Divorced	2	6.3	9	29.0	
Widowed	11	34.4	6	19.4	
Education					
No formal education	11	36.7	14	45.2	0.189
Less than fifth grade	7	23.3	1	3.2	
5th–8th grade	6	20.0	8	25.8	
8th–10th grade	2	6.7	1	3.2	
10th–12th grade	1	3.3	3	9.7	
Completed high school	0	0.0	2	6.5	
More than high school	3	10.0	2	6.5	
Income					
<$25,000	24	80.0	25	80.7	1.00
$25,000–49,999	4	13.3	5	16.1	
$50,000–74,999	1	3.3	0	0.0	
$75,000–99,999	1	3.3	0	0.0	
Prefer not to answer	0	0.0	1	3.2	
Primary language					
Somali	31	100.0	31	100.0	–
Need an interpreter					
No	6	19.4	3	9.7	0.473
Yes	25	80.7	28	90.3	

Table2 shows the health-related characteristics of participants by treatment group. More than half of the participants never had a Pap test, although this differed by randomization group (*n* = 12, 38.7% in clinic group vs. *n* = 20, 62.5% in home group; *P* = 0.062). The majority of women reported having health insurance and a regular doctor and did not think they were at risk of cervical cancer. More than half of the women reported having more than four previous pregnancies. All but two (96.6%) of the women reported that their doctors had never discussed with them anything to do with HPV vaccine. Beyond timing of last Pap test, there was no evidence of differences between randomized groups on these characteristics.

**Table 2:** Health-related characteristics (*n* = 63)

Characteristic	Clinic-based Pap group		Home-based HPV kit group		*P*-value
	*N*	%	*N*	%	
Last Pap test					
3–5 years	14	45.2	8	25.0	0.062
5–10 years	2	6.5	4	12.5	
>10 years	3	9.7	0	0.0	
Never	12	38.7	20	62.5	
Regular doctor					
No	6	19.4	3	9.7	0.473
Yes	25	80.7	28	90.3	
Health care coverage					
No	4	12.9	3	9.7	1.00
Yes	27	87.1	28	90.3	
Time since last general exam					
Within past 2 years	25	80.7	25	80.7	0.776
More than 2 years	4	12.9	3	9.7	
Never	1	3.2	3	9.7	
Don't Know	1	3.2	0	0.0	
Doctor visits last year					
None	15	48.4	10	32.3	0.410
1–4 times	10	32.3	12	38.7	
5 + times	6	19.4	9	29.0	
Difficult to get primary care clinic					
No	29	96.7	28	90.3	0.612
Yes	1	3.3	3	9.7	
Prefer female provider					
Strongly disagree	1	3.3	2	6.5	0.514
Somewhat disagree	2	6.7	4	12.9	
Somewhat agree	1	3.3	3	9.7	
Strongly agree	26	86.7	22	71.0	
Difficult to get interpreter for health care					
Strongly disagree	25	83.3	25	80.7	1.00
Somewhat disagree	1	3.3	1	3.2	
Somewhat agree	0	0.0	0	0.0	
Strongly agree	4	13.3	5	16.1	
Want to know chance of getting cancer					
Strongly disagree	11	36.7	17	54.8	0.286
Somewhat disagree	2	6.7	2	6.5	
Somewhat agree	0	0.0	1	3.2	
Strongly agree	17	56.7	11	35.5	
Think you are at risk for cervical cancer					
Strong disagree	21	70.0	28	90.3	0.130
Somewhat disagree	6	20.0	2	6.5	
Somewhat agree	2	6.7	0	0.0	
Strongly agree	1	3.3	1	3.2	
See benefit in cancer screening					
Strong disagree	1	3.3	2	6.5	1.00
Somewhat disagree	0	0.0	0	0.0	
Somewhat agree	0	0.0	1	3.2	
Strongly agree	29	96.7	28	90.3	
History of C-section					
No	28	93.3	28	90.3	1.00
Yes	2	6.7	3	9.7	
History of female circumcision					
No	2	6.7	4	12.9	0.671
Yes	28	93.3	27	87.1	
Number of previous pregnancies					
None	4	13.3	3	9.7	0.767
1–2	3	10.0	3	9.7	
3–4	3	10.0	6	19.4	
More than 4	20	66.7	19	61.3	
Friends/family members to talk about health					
No	11	36.7	10	32.3	0.717
Yes	19	63.3	21	67.7	
Friends/family members to talk about cancer screening					
No	21	70.0	16	51.6	0.142
Yes	9	30.0	15	48.4	
Anyone in family ever had cancer					
No	26	86.7	24	77.4	0.508
Yes	4	13.3	7	22.6	
Ever told by doctor to have mammogram					
No	11	36.7	10	32.3	0.717
Yes	19	63.3	21	67.7	
Doctor ever discussed HPV vaccine with you?					
No	29	100.0	27	93.1	0.491
Yes	0	0.0	2	6.9	
Feel comfortable sending testing materials in mail					
No	13	43.3	12	38.7	0.714
Yes	17	56.7	19	61.3	
Prefer to receive medical test results					
In-person	7	23.3	3	9.7	0.262
Mail	20	66.7	22	71.0	
Phone	3	10.0	6	19.4	

Analysis of the primary outcome indicated that participants randomized to the home test group were more likely to complete the test (21/32, 65.6%) than those randomized to the clinic group (6/31, 19.4%); *P* = 0.0002). Univariate logistic regression analyses indicated that those randomized to the home group, those who reported having family/friends to talk about cancer screening, and those who have been in the US longer were more likely to have completed screening (Table3). After adjusting for time since last Pap test, whether friends/family members talk about cancer screening and number of years in the US, multivariate logistic regression analyses indicated that women randomized to the home-based HPV group were about 14 times more likely to complete screening than those assigned to the clinic-based Pap group (OR: 14.18 [95% CI: 2.73–73.51]). While this was highly statistically significant (*P* = 0.002), this odds ratio should be cautiously interpreted given the sample size and large confidence interval.

**Table 3:** Factors associated with test completion (*n* = 63)

Variable	Test completed		Univariate analysis		Multivariate analysis^1^	
	*N*	%	OR (95% CI)	*P*-value	OR (95% CI)	*P*-value
Randomization group						
Clinic	6	19.4	1.00	0.0004	1.00	0.002
Home	21	65.6	7.95 (2.52–25.16)		14.18 (2.73–73.51)	
Last Pap test						
3–5 years	10	45.5	1.00	0.949	1.00	0.973
5–10 years	3	50.0	1.20 (0.20–7.31)		1.43 (0.05–44.80)	
>10 years	1	33.3	0.60 (0.05–7.63)		1.64 (0.11–25.30)	
Never	13	40.6	0.82 (0.27–2.46)		0.86 (0.16–4.73)	
Marital status						
Single/never	2	33.3	0.44 (0.06–3.11)	0.706		
Married	8	36.4	0.51 (0.14–1.84)			
Widowed	8	47.1	0.79 (0.21–3.04)			
Divorced/separated	9	52.9	1.00			
Education						
No formal education	9	36.0	1.00	0.525		
Less than high school	12	48.0	1.64 (0.53–5.09)			
Completed high school or more	6	54.6	2.13 (0.51–9.01)			
Regular doctor						
Yes	21	39.6	0.33 (0.07–1.46)	0.143		
No	6	66.7	1.00			
Friends/family members to talk about cancer screening						
Yes	15	62.5	3.47 (1.18–10.18)	0.023	3.14 (0.72–13.67)	0.127
No	12	32.4	1.00		1.00	
Want to know chance of getting cancer						
Strongly agree/somewhat agree	17	53.1	0.46 (0.17–1.31)	0.146		
Strongly disagree/somewhat disagree	10	34.5	1.00			
Anyone in family ever had cancer						
Yes	7	63.6	2.63 (0.68–10.15)	0.162		
No	20	40.0	1.00			
Age (per 5 year increase)	27		0.98 (0.81–1.18)	0.804		
Years in United States (per 1 year increase)	27		1.16 (1.04–1.28)	0.007	1.23 (1.05–1.44)	0.011

1 Adjusted for randomization group, time since last Pap test, whether friends/family members to talk about cancer screening and number of years in the United States.

Women who reported having friends/family members to talk about cancer screening were approximately three times more likely to complete screening than those who do not, although this was not statistically significant after multivariate adjustment (OR: 3.14; [95% CI: 0.72–13.67], *P* = 0.127). Finally, women who have lived in the US longer were more likely to complete screening remained statistically significant after adjustment (OR per 1 year longer: 1.23; [95% CI: 1.05–1.44], *P* = 0.011).

The results from the acceptability survey completed by women in the HPV test group showed that 83% of these women indicated that if they had to make a choice between using the self- collection and the regular Pap test, they would choose the self-collection method. All participants indicated that the instructions were easy to follow and had no difficulties in collecting the sample. In addition, 30% of women agreed to DNA sample storage.

HPV genotyping results showed that 3/21 (14.2%) of women who completed and returned the home-based HPV test kit had a positive result; two participants were positive for HPV 16 while one participant was positive for HPV 68; both HPV types are considered high risk for cervical cancer. Test results for all the 21 women will be delivered and they will be strongly encouraged to obtain a clinic-based Pap test as this is the current standard of care in the US. Results from the clinic-based Pap test obtained from six participants were all negative for intraepithelial lesion or malignancy.

## Discussion

This pilot study compares HPV home-based cervical cancer screening rates versus clinic-based Pap test among Somali immigrant women in the US. We found a screening completion rate of 65.6% for the home-based HPV kit versus 19.4% for the clinic-based Pap test. The screening completion rates in our study are comparable to findings from a systemic review and meta-analysis that showed that the overall relative compliance of HPV self-collected testing was significantly greater compared to Pap testing 37. Several other studies have shown a high acceptability rate for the home-based self-sampling over Pap test 38–42. However, some studies have assessed the barriers to acceptance of the self-sampling HPV test and showed that some women preferred Pap testing by a health care professional because they were accustomed to pelvic examinations, the test was more convenient or they trusted the results 15,43–45.

Furthermore, the Pap test completion rates for Somali women in our study were low at 19.4% compared to those reported in a previous study done by Morrison and colleagues in Minnesota, where the Pap test completion rates for Somali immigrant women were at 48.8% 13. This discrepancy could be a result of two very different recruitment strategies: Morrison et al. used a clinic data base for his study, while our study recruited participants who may or may not have been enrolled in a clinic, an approach which could have created the potential for such a difference. A larger size study is needed to validate these findings. Our findings further confirm that foreign-born women are less likely to receive a Pap test compared to US born women 6.

In addition, our study showed that women who reported having a social network of friends and family to talk about cancer screening and had more than 10 years of residency in the US were more likely to complete the screening test. This information further justifies the need for a community-based model that involves friends and family members in programs designed to increase screening utilization in immigrant communities. This implies that an intervention targeting the individual and their support system may enhance cervical cancer screening among under screened women. Our findings are similar to those from studies finding that one of the strong factors associated with cervical cancer screening among immigrants is suggestion from friends, followed by longer residency 9,46–48.

A large number of our study participants were already engaged in the health care system, with more than 80% having had a general physical exam within the past 2 years. The majority reported visiting a regular health care provider and had no difficulty in getting to their primary care clinic. Despite their encounters in the health care environment, more than 50% reported never receiving a Pap test. These patient clinic encounters should be used as opportunities to expand alternative methods of cervical cancer screening within the current clinic setting.

Lack of insurance coverage is considered a significant barrier to cancer screening 49; however, in our study, the majority of women reported having health insurance. More than 50% of the women reported that they were not at risk for cervical cancer and nearly 50% did not want to know their chances of getting cancer. This supports other studies highlighting the significance of increasing awareness as component to any cervical cancer prevention initiative among immigrant communities 14.

Studies have documented several sociocultural factors that influence cervical cancer screening among immigrants and ethnic minorities. Commonly held beliefs cut across several cultural groups 50–52. One unique finding in our study is that more than 80% of our study participants strongly believed that they were not at any risk of getting cervical cancer, which is in agreement with the HPV positivity test results we obtained. Our findings are similar to the perceptions and barriers that have been well documented showing that many Somali women held fatalistic attitudes and lacked an understanding of the risk factors for cervical cancer 14,51. The strong belief that one is not at risk of a getting a particular disease has been documented as a deterrent for an individual to make an effort to undergo screening. The health belief model framework shows that individuals who believe they are at no risk are less likely to screen for a disease 53.

This study has some limitations. First, it was conducted in a metropolitan area in the upper Midwest of the US and there may be differences between cities, which limits the external validity. However, this generalizability concern is somewhat mitigated by the fact that Minneapolis has the largest Somali immigrant population in the US. In addition, this was a pilot study and therefore the sample size used here was small. Due to the small sample size and random chance, the randomization did not completely balance the two groups for all potential confounders. In this case, there was a difference in the proportion who had never had a pap test before between the groups (*n* = 20 (63%) in the home group versus *n* = 12 (39%) in the pap group). We attempted to account for this difference in the analysis but this could potentially be avoided in future studies by either enrolling a larger sample and/or by stratifying the randomization on this variable. Finally, we solely relied on self-report of Pap test history; this may have created bias if some of the participants were not aware if they had the screening test within the past 3 years.

This study adds to the literature by providing evidence that despite the existing barriers to cancer screening in the Somali immigrant community, women are willing and able to use an alternative screening test as an initial approach to cervical cancer screening.

## Conclusion

The use of a self-sampling HPV kit has the potential to increase screening in this community; it addresses some major sociocultural barriers such as lack of privacy created by the need for an interpreter during a clinic-based Pap test. Future research should explore using the home-based HPV test kits as an initial approach to encourage use of cervical cancer screening in the Somali community. In addition, we need to develop culturally and linguistically relevant screening education programs that address the sociocultural factors that continue to fuel the growing cervical cancer screening disparities in immigrants and minority ethnic populations in the US.
